# Impact of prolonged assisted ventilation on diaphragmatic efficiency: NAVA *versus* PSV

**DOI:** 10.1186/s13054-015-1178-0

**Published:** 2016-01-05

**Authors:** Rosa Di mussi, Savino Spadaro, Lucia Mirabella, Carlo Alberto Volta, Gabriella Serio, Francesco Staffieri, Michele Dambrosio, Gilda Cinnella, Francesco Bruno, Salvatore Grasso

**Affiliations:** 1 Dipartimento dell’Emergenza e Trapianti d’Organo (DETO), Sezione di Anestesiologia e Rianimazione, Università degli Studi di Bari “Aldo Moro”, Piazza Giulio Cesare 11, Bari, Italy; 2 Dipartimento di Morfologia, Chirurgia e Medicina Sperimentale, Sezione di Anestesiologia e Terapia Intensiva Universitaria, Università degli studi di Ferrara, Ferrara, Italy; 3 Dipartimento di Anestesia e Rianimazione, Università di Foggia, Foggia, Italy; 4 Dipartimento di Scienze Biomediche ed Oncologia Umana, Cattedra di Statistica Medica, Università degli Studi Aldo Moro, Bari, Italy; 5 Dipartimento dell’Emergenza e Trapianti d’Organo (DETO), Sezione di Chirurgia Veterinaria, Università degli Studi di Bari “Aldo Moro”, Bari, Italy

**Keywords:** Mechanical ventilation, Assisted modes of ventilation, Neurally adjusted ventilatory assist (NAVA), Pressure support ventilation (PSV)

## Abstract

**Background:**

Prolonged controlled mechanical ventilation depresses diaphragmatic efficiency. Assisted modes of ventilation should improve it. We assessed the impact of pressure support ventilation *versus* neurally adjusted ventilator assist on diaphragmatic efficiency.

**Method:**

Patients previously ventilated with controlled mechanical ventilation for 72 hours or more were randomized to be ventilated for 48 hours with pressure support ventilation (n =12) or neurally adjusted ventilatory assist (n = 13). Neuro-ventilatory efficiency (tidal volume/diaphragmatic electrical activity) and neuro-mechanical efficiency (pressure generated against the occluded airways/diaphragmatic electrical activity) were measured during three spontaneous breathing trials (0, 24 and 48 hours). Breathing pattern, diaphragmatic electrical activity and pressure time product of the diaphragm were assessed every 4 hours.

**Results:**

In patients randomized to neurally adjusted ventilator assist, neuro-ventilatory efficiency increased from 27 ± 19 ml/μV at baseline to 62 ± 30 ml/μV at 48 hours (p <0.0001) and neuro-mechanical efficiency increased from 1 ± 0.6 to 2.6 ± 1.1 cmH_2_O/μV (p = 0.033). In patients randomized to pressure support ventilation, these did not change. Electrical activity of the diaphragm, neural inspiratory time, pressure time product of the diaphragm and variability of the breathing pattern were significantly higher in patients ventilated with neurally adjusted ventilatory assist. The asynchrony index was 9.48 [6.38– 21.73] in patients ventilated with pressure support ventilation and 5.39 [3.78– 8.36] in patients ventilated with neurally adjusted ventilatory assist (p = 0.04).

**Conclusion:**

After prolonged controlled mechanical ventilation, neurally adjusted ventilator assist improves diaphragm efficiency whereas pressure support ventilation does not.

**Trial registration:**

ClinicalTrials.gov study registration: NCT0247317, 06/11/2015.

## Background

In the acute phase of critical illness, controlled mechanical ventilation (CMV) improves gas exchange and alleviates respiratory fatigue [1]. On the other hand, CMV induces diaphragmatic atrophy [2], decreases diaphragmatic efficiency [2–4], requires deep sedation and even paralysis, and causes lung atelectasis [5]. Expert opinion and clinical guidelines recommend shifting as soon as possible from CMV to modes in which the ventilator applies positive pressure at the airway opening to support the patient’s spontaneous inspiratory effort [6, 7].

During pressure support ventilation (PSV), the most commonly used assisted mode [8], the ventilator applies a constant (operator set) level of positive pressure throughout the inspiratory phase [9, 10]. The expiratory phase begins when the inspiratory flow decays below a predefined threshold. The interplay between inspiratory effort, level of assistance and impedance of the respiratory system determines the instantaneous inspiratory flow [7]. Though PSV efficiently unloads respiratory muscles [10], it delivers a fixed assistance. In other terms, PSV does not affect the neuro-ventilatory coupling [11, 12]. Furthermore, during PSV a high incidence of patient-ventilator asynchronies may occur [13, 14].

Neurally adjusted ventilatory assist (NAVA) is a recently introduced mode based on the measurement of electrical activity of the diaphragm (EAdi) [15, 16]. Briefly, in the inspiratory phase the ventilator delivers positive pressure in proportion to EAdi and cycling from the inspiratory to the expiratory phase occurs when the EAdi decays below a predefined threshold. In NAVA neural and mechanical inspiratory times are better synchronized than in PSV [12]. By delivering a proportional assistance, NAVA improves the neuro-ventilatory coupling [11, 12]. Several studies have shown a lower incidence of patient-ventilator asynchronies in NAVA as compared to PSV [17–19].

In this study we tested the impact of PSV versus NAVA on diaphragmatic efficiency, expressed in terms of neuro-ventilatory and neuro-muscular efficiency (NVE and NME, respectively). The NVE outlines the diaphragmatic ability to convert EAdi into inspired volume, while the NME outlines the ability to convert EAdi into inspiratory pressure [19–21]. We randomly assigned patients previously ventilated in CMV for at least 72 hours to be ventilated with PSV or NAVA for the following 48 hours. The NVE and NME were measured during three brief spontaneous breathing trials, at 0, 24 and 48 hours. We hypothesized that after a CMV period potentially able to depress diaphragm efficiency [2–4, 22], prolonged assisted ventilation would improve NVE and NME, and that in this context, NAVA would be superior to PSV owing to its more physiological algorithm of assistance.

## Methods

### Patient selection

Patients admitted over a period of one year (from May 2013 to May 2014) to the ICU of the University of Bari Academic Hospital were considered for enrollment in the study. The local ethics committee (Azienda Ospedaliero-Universitaria Policlinico di Bari Ethic Committee, protocol number: 257/C.E. March 2013) approved the investigative protocol, and written informed consent was obtained from each patient or next of kin. A physician not involved in the study was always present for patient care. Our clinical trial was registered with clinicalTrials.gov, identifier: NCT02473172.

Patients were eligible for the study if they were older than 18 years, oro-tracheally or naso-tracheally intubated, had been ventilated for acute respiratory failure with CMV (flow-limited, pressure-limited or volume-targeted pressure-limited) for at least 72 hours consecutively and were candidates for assisted ventilation. The criteria for defining the readiness to assisted ventilation were: a) improvement of the condition leading to acute respiratory failure; b) positive end-expiratory pressure (PEEP) lower than 10 cmH_2_O and inspiratory oxygen fraction (FiO_2_) lower than 0,5; c) Richmond agitation sedation scale (RASS) score between 0 and –1 [23] obtained with no or moderate levels of sedation and, d) ability to trigger the ventilator, i.e., to decrease pressure airway opening (P_AO_) >3–4 cmH_2_O during a brief (5–10 s) end-expiratory occlusion test. Other criteria included hemodynamic stability without vasopressor or inotropes (excluding a dobutamine and dopamine infusion <5 gamma/Kg/min and 3 gamma/Kg/min, respectively) and normothermia. Patients were excluded from the study if they were affected by neurological or neuromuscular pathology and/or known phrenic nerve dysfunction, or if they had any contraindication to the insertion of a nasogastric tube (for example: recent upper gastrointestinal surgery, esophageal varices).

### Measurements

Patients were studied in the semi-recumbent position. All patients were ventilated with a Servo i ventilator (Maquet Critical Care, Solna, Sweden) equipped with the NAVA software. At the beginning of the study the standard naso-gastric tube was replaced with a 16 Fr, 125 cm, EAdi catheter (Maquet Critical Care, Solna, Sweden). The EAdi catheter was first positioned according to the corrected nose-ear lobe-xyphoid distance formula [24]. Its position was subsequently titrated through the EAdi catheter position tool (Servo i, NAVA software) [24]. The digital P_AO_, Flow and EAdi signals obtained from the RS232 port of the ventilator were stored in a personal computer at a sampling rate of 100 Hz (NAVA tracker software, Maquet Critical Care, Solna, Sweden). Subsequently the NAVA tracker files were converted and analyzed using the ICU Lab software package (Kleistek Engineering; Bari, Italy).

Peak airway opening pressure (P_AO PEAK_) and PEEP were measured from the P_AO_ signal. Tidal volume (VT) was measured as the integral of the inspiratory flow. Mechanical respiratory rate (RR_MECH_) was measured by the flow and P_AO_ signals. Mechanical inspiratory and expiratory time (Ti,_MECH_ and Te,_MECH_, respectively) were determined from the flow signal. Peak EAdi (EAdi_PEAK_) and neural inspiratory time (Ti,_NEUR_) were determined from the EAdi signal [19].

The NVE was calculated as: VT/EAdi_PEAK_; the NME was calculated as the ratio between the peak negative value in airway pressure of a single inspiratory effort (recorded during a 2–3 s end-expiratory occlusion) and the corresponding EAdi_PEAK_ [19–21].

The pressure generated by the diaphragm (P_DI_) was calculated from the EAdi signal according to Bellani and coworkers [25]. Briefly, since the fall in P_AO_ during a spontaneous inspiratory effort against the occluded airways is equal to the fall in esophageal pressure (P_ES_) [26, 27], the NME can be used as an index to convert the EAdi signal into a P_DI_ signal:

$$ {\mathrm{P}}_{\mathrm{DI}}=\mathrm{EAdi}*\mathrm{N}\mathrm{M}\mathrm{E} $$

The inspiratory pressure–time product of the P_DI_ per breath (PTP_DI_/b) was calculated as the area under the P_DI_ signal. The PTP_DI_ per minute (PTP_DI_/min) was calculated as:

$$ \mathrm{P}\mathrm{T}{\mathrm{P}}_{\mathrm{DI}}/ \min =\mathrm{P}\mathrm{T}{\mathrm{P}}_{\mathrm{DI}}/\mathrm{b}*\mathrm{R}\mathrm{R}. $$

The coefficient of variation (CV) for breathing pattern and the EAdi parameters was calculated as: standard deviation/mean.

### Study protocol

At the beginning of the study, patients were randomized to the PSV or NAVA mode. The PEEP and FiO_2_ levels that were in use during CMV were left unchanged.

In patients randomized to the PSV mode, the inspiratory pressure level was titrated to obtain a VT between 5 and 8 ml/Kg predicted body weight (PBW) [10, 19, 28]. The inspiratory trigger was set in the flow-by mode, at a sensitivity level of 5 (Servo i arbitrary units), unless a higher level was required to avoid auto-triggering; the expiratory trigger was set at 30 % of the peak inspiratory flow. In patients randomized to the NAVA mode, the NAVA level was titrated according to Brander and coworkers [29, 30]. Briefly, the NAVA level was reduced to zero and subsequently increased by 0.1 cmH_2_O/μV every 20 s while observing the time/plot of P_AO_ and VT on the ventilator screen. The progressive increase in assistance during NAVA generates an initial steep increase in P_AO_ and VT (first response) followed by a less steep increase, or plateau (second response); the optimal NAVA level is early after the transition from the first to the second response [30]. During NAVA, the ventilator assist can be triggered in two different ways according to the first come, first served principle: a) EAdi-based neural trigger and, b) flow or pressure pneumatic trigger. In the latter case, the ventilator applies an initial inspiratory pressure of 2 cmH_2_O and thereafter the inspiratory pressure is guided by the EAdi signal. The pneumatic trigger was set with the same criteria as for PSV; the EAdi trigger was set at a 0.5 μV threshold (unless a higher level was required to avoid auto-triggering). The NAVA inspiratory to expiratory cycling-off is by default at the 70 % of the preceding EAdi_PEAK_.

Throughout the study the attending physicians were allowed to change the NAVA or PSV settings or the ventilator mode, if appropriate for clinical reasons. In case of change of ventilatory mode, the patient was dropped from the study. The reasons to suspend the assigned mode were recorded on the study database. According to our clinical protocol for assisted ventilation, throughout the study the patients were kept unsedated or moderately sedated (RASS between 0 and –1) by using no sedatives or moderate doses of remifentanil and/or midazolam and/or propofol, as clinically indicated.

During the study, the patients underwent three brief (2 minutes) of spontaneous breathing trials (SBT): a) immediately before shifting to the randomized assisted mode (0 hours); b) after 24 hours and c) after 48 hours of assisted ventilation. Briefly, the ventilatory mode was shifted to continuous positive airway pressure (CPAP), at the same level of PEEP and FiO_2_ used in the assisted mode. At the end of the SBT, a brief (2–3 s) end-expiratory occlusion (appropriate knob of the Servo i ventilator) was realized to measure the NME. Immediately before each SBT the sedation level was assessed through the RASS score and arterial blood gas analysis was obtained. Patients were immediately reconnected to the ventilator and dropped from the study if during the SBT they showed any of the following signs of respiratory or cardiovascular distress: a) paradoxical abdominal motion or other signs of accessory respiratory muscle and/or other signs of respiratory muscle fatigue; b) cardiovascular instability (Pas >160 or <90 mmHg or 20 % different from the pre-SBT values; heart rate (HR) >120 or <60 beat/minutes or 20 % different from the pre-SBT values); c) arterial desaturation with arterial hemoglobin saturation (SaO_2_ )< 94 % or SaO_2_ decrease of more than 2–3 % from the pre-SBT values.

### Data analysis

Breathing pattern, EAdi-derived parameters and patient-ventilator asynchronies were calculated offline from the P_AO_, flow and EAdi digital recordings. Breathing pattern analysis was performed on the entire SBT periods and on the last 60 breaths of each 4-hour period. Patient-ventilator asynchronies were detected on the last 10 minutes of each 4-hour period (total 120 minutes) by offline visual inspection of P_AO_, flow and EAdi recordings [19] by three of the authors, SG, SS, CAV, with specific expertise in the field of patient-ventilator interactions. According to Thille and coworkers, the asynchronies were classified into six types: a) ineffective triggering (missed effort); b) ineffective inspiratory triggering; c) double-triggering; d) auto-triggering; e) prolonged cycle; f) short cycle [13]. The asynchrony index (AI) was calculated as:

$$ \mathrm{AI}=\mathrm{Total}\kern0.5em \mathrm{number}\kern0.5em \mathrm{of}\kern0.5em \mathrm{asynchronies}/\left(\mathrm{mechanical}\kern0.5em \mathrm{cycles}+\mathrm{missed}\kern0.5em \mathrm{efforts}\right). $$

### Statistical analysis

The normal distribution of quantitative data was evaluated through the D’Agostino test. All the data approaching the normal distribution are summarized as mean and standard deviation (SD). Non-normally distributed data are expressed as median (interquartile range).

Breathing pattern, gas exchange and EAdi-derived parameters at the three SBT time points (0, 24 and 48 hours) were normally distributed and differences were evaluated through an analysis of variance (ANOVA) model for repeated measure with interaction (time*method). Post hoc comparisons, between the two groups at each time point and within each group between the three time points, were carried out using Student’s *t* test, with Bonferroni correction. The significance level for the ANOVA model was set at 0.05 whereas the significance level for post-hoc comparisons was set at 0.006.

The breathing pattern and EAdi-derived parameters measured every 4 hours throughout the study (12 points for each variable) were normally distributed. Their trends were compared through an ANOVA model for repeated measures, with an effect between methods (NAVA vs PSV), an effect within subject and an interaction term. Statistical significance for this model was set at 0.05. The total number of each asynchrony type, the AI and the CV of the breathing pattern parameters were not normally distributed. Their differences were evaluated though the Mann–Whitney test; the significance level was set at 0.05.

## Results

**Fig. 1:**
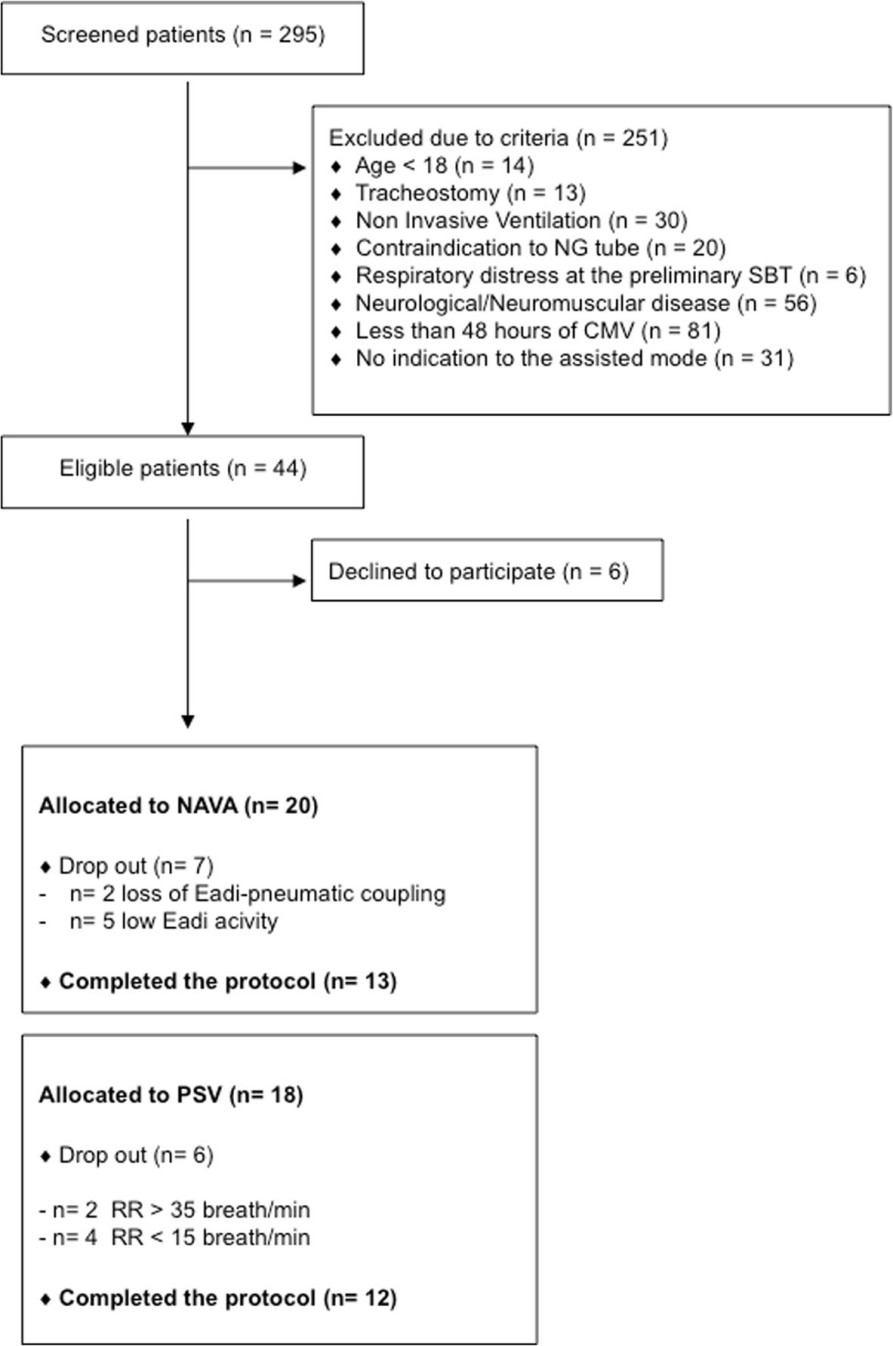
Flow diagram of patient enrollment. *NG* naso-gastric; *SBT* spontaneous breathing trial, *CMV* controlled mechanical ventilation, *NAVA* adjusted ventilatory assist, *EAdi* diaphragm electrical activity, *PSV* pressure support ventilation, *RR* respiratory rate

Of the 44 eligible patients, 38 were enrolled (6 declined to participate): 20 were randomized to NAVA and 18 to PSV (Fig. 1). A reliable EAdi signal was obtained in all the patients at baseline. In the NAVA group, 7 out of 20 patients (35 %) did not complete the protocol according to the decision of the attending physician, 2 for loss of EAdi-pneumatic synchrony, 5 for EAdi signal persistently lower than the EAdi trigger threshold. In the PSV group, 6 out of 18 patients (33 %) did not complete the protocol according to the decision of the attending physician: 2 for persistently high respiratory rate (RR) (i.e., RR >35 breaths/minute) and 4 for persistently low RR (i.e., RR <15 breaths/minute). No patient was dropped from the study because of distress during any of the SBTs. The demographical and clinical characteristics of the studied patients are shown in Table 1.

**Table 1:** Baseline demographic and clinical characteristics of the patients

	Patient number	Age	Sex	PBW (Kg)	APACHE II	Causes of ARF	MV (days)	NAVA or PSV level (μV or cmH_2_O)	PEEP (cmH_2_O)	FiO_2_ %	ICU outcome
NAVA group	1	65	M	70	12	Politrauma	5	1	8	35	A
	2	54	M	75	14	ARDS, sepsis	4	2	5	40	A
	3	79	M	61	9	Politrauma	3	1,4	5	40	A
	4	83	F	69	23	Pneumonia	5	1,2	6	50	D
	5	27	F	48	10	Cardiac Failure	6	1,8	6	35	A
	6	72	F	73	7	COPD exacerbation	4	1	8	50	A
	7	83	F	60	8	Politrauma	7	1,5	8	35	A
	8	63	M	67	16	Cardiac failure	4	1	10	50	D
	9	47	F	57	15	ARDS, pneumonia	4	1,2	9	40	A
	10	81	M	75	13	COPD exacerbation	7	1,5	6	45	A
	11	81	F	52	15	Cardiac failure	4	2	8	50	A
	12	80	F	61	13	COPD exacerbation	4	1	8	40	A
	13	54	F	58	17	Pneumonia	9	1	10	50	D
**Mean**		**66.8**		**63.5**	**13.2**		**5.1**	**1.35**	**7.5**	**43.1**	
**SD**		**17.3**		**8.7**	**4.3**		**1.7**	**0.38**	**1.7**	**6.3**	
PSV group	1	76	M	70	11	Politrauma	5	12	8	40	A
	2	80	M	66	14	Pneumonia	6	12	8	40	A
	3	64	M	61	10	ARDS, politrauma	4	11	8	55	A
	4	77	M	66	13	Pancreatitis	4	8	5	40	D
	5	28	F	43	12	SLE	5	14	8	35	A
	6	75	M	69	12	COPD exacerbation	6	12	9	60	A
	7	74	F	52	20	Pneumonia, ARDS	4	11	6	50	A
	8	84	F	66	17	Politrauma	8	15	7	45	A
	9	65	M	62	20	ARDS, politrauma	7	10	10	50	D
	10	73	M	70	13	ARDS, sepsis	4	8	10	35	A
	11	81	M	68	15	Cardiac Failure	4	12	8	60	A
	12	61	M	66	18	COPD exacerbation	5	12	8	35	D
**Mean**		**69.8**		**63.2**	**14.6**		**5.1**	**11.4**	**7.9**	**45.4**	
**SD**		**15**		**8.1**	**3.4**		**1.3**	**2.1**	**1.4**	**9.4**	

*NAVA* neurally adjusted ventilator assist, *PSV* pressure support ventilation, *PBW* predicted body weight, *APACHE II*, Acute Physiology, Age and Chronic Health Evaluation II score (can range from 0 to 299, with higher scores indicating a higher probability of death), *ARF* acute respiratory failure, *MV* mechanical ventilation, *PEE*P positive end-expiratory pressure, *ARDS* acute respiratory distress syndrome, *SLE* systemic lupus erythematosus, *COPD* chronic obstructive pulmonary disease, *FiO*_*2*_ inspiratory oxygen fraction, *D* death, *A* alive

### Spontaneous breathing trials

**Fig. 2:**
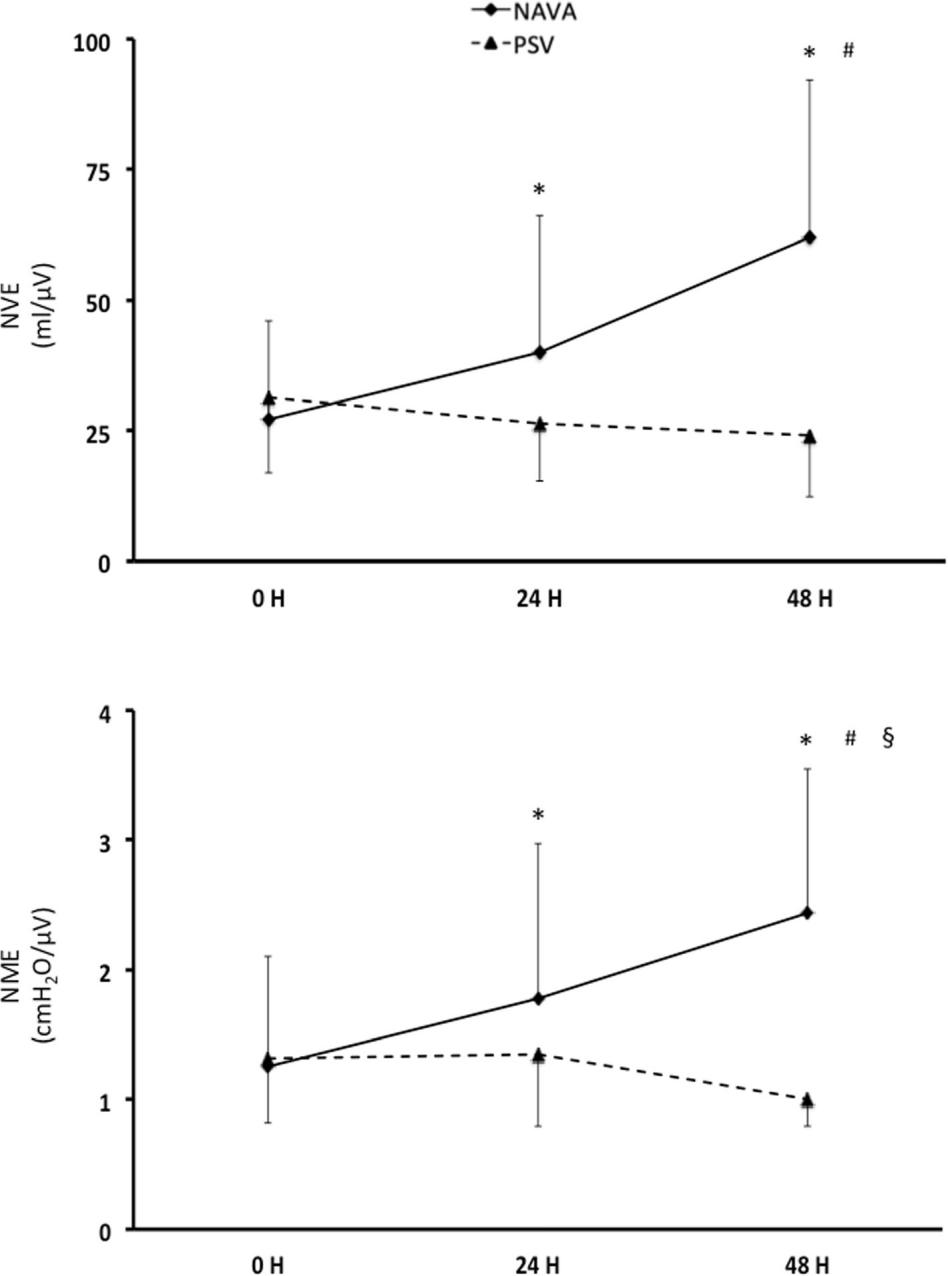
Neuro-ventilatory efficiency (*NVE*) and neuro-muscular efficiency (*NME*) recorded during the spontaneous breathing trial at 0, 24 and 48 hours in the two groups of patients, i.e., randomized to be ventilated in neurally adjusted ventilatory assist (*NAVA*) or pressure support ventilation (*PSV*). The NVE and NME trends were significantly different (analysis of variance model; *F* = 15.32; *p* <0,0001 for NVE and *F* = 5,15; *p* = 0.033 for NME). *Significant difference compared to 0 hours (within-group post-hoc comparison). ^#^Significant difference compared to 24 hours (within-group post-hoc comparison). ^§^Significant difference compared to the same time (between-groups post-hoc comparison)

The ANOVA model showed a significant difference in the NVE and NME trend between the groups (*F* = 15.32; *p* <0,0001 for NVE and *F* = 5,15; *p* = 0,033 for NME) (Fig. 2). While NVE and NME were similar between the groups at baseline, at 48 hours both were significantly higher in the NAVA than in the PSV group (Fig. 2).

Table 2 shows the breathing pattern and EAdi parameters recorded during the three SBTs and, in addition, the RASS score and the gas exchange parameters recorded immediately before each SBT.

**Table 2:** Breathing pattern and EAdi parameters during the SBTs and gas exchange parameters immediately before each SBT

	PSV group			NAVA group		
	0 h	24 h	48 h	0 h	24 h	48 h
CPAP (cmH_2_O)	8.43 ± 2.4	8.02 ± 2.8	8.09 ± 1.54	8.3 ± 2.3	8 ± 2.7	7.8 ± 2.8
VT (ml)	404 ± 185	413 ± 154	412 ± 172	378 ± 124	375 ± 120	408 ± 120
RR (breaths/minute)	25.9 ± 12.2	23.7 ± 9.1	23.7 ± 6.7	24.3 ± 6.5	24 ± 7.8	23.6 ± 8.4
Ti,_NEUR_ (s)	0.94 ± 0.36	0.93 ± 0.33	0.93 ± 0.26	0.87 ± 0.25	0.80 ± 0.18	0.82 ± 0.39
Flow peak (L/s)	0.60 ± 0.2	0.59 ± 0.1	0.55 ± 0.11	0.66 ± 0.27	0.68 ± 0.3	0.66 ± 0.2
EAdi_PEAK_ (μV)	14.2 ± 6.2	17.2 ± 6.7*	19.06 ± 9.2*	18.9 ± 10.1	13.2 ± 8.9*	9.2 ± 5.5*§
pH	7.41 ± 0.1	7.41 ± 0.1	7.44 ± 0.1	7.46 ± 0.1	7.46 ± 0.1	7.45 ± 0.1
PaO_2_/FiO_2_	214 ± 70	231 ± 86	231 ± 93	239 ± 75	260 ± 75	289 ± 72
PaCO_2_ (mmHg)	46.3 ± 12.4	46 ± 11.5	45.6 ± 9.9	45.5 ± 11.4	46.4 ± 11.1	47.6 ± 14.6
HCO3^−^ (mEq/L)	29.43 ± 6.2	29.7 ± 6.3	30.6 ± 7.2	31.5 ± 5.5	33 ± 5.6	32.6 ± 7.9
RASS score	–0.5 ± 1	–0.33 ± 1.15	–0.25 ± 1.14	–0.38 ± 1.04	–0.46 ± 1.13	– 0.54 ± 1.05

*p <0,006 versus 0 h, same group; ^§^ *p* <0,006 between groups, same time point. *EAdi* diaphragm electrical acivity, *SBT* spontaneous breathing trial, *NAVA* neurally adjusted ventilator assist, *PSV* pressure support ventilation, *CPAP* continuous positive airway pressure *VT* tidal volume, *RR* respiratory rate, *Ti,*_*NEUR*_ neural inspiratory time, *PaO*_*2*_ arterial oxygen partial pressure, *FiO*_*2*_ inspiratory oxygen fraction, *PaCO*_*2*_ arterial carbon dioxyde partial pressure, *RASS* Richmond agitation sedation scale [23]

### Assisted ventilation periods

**Fig. 3:**
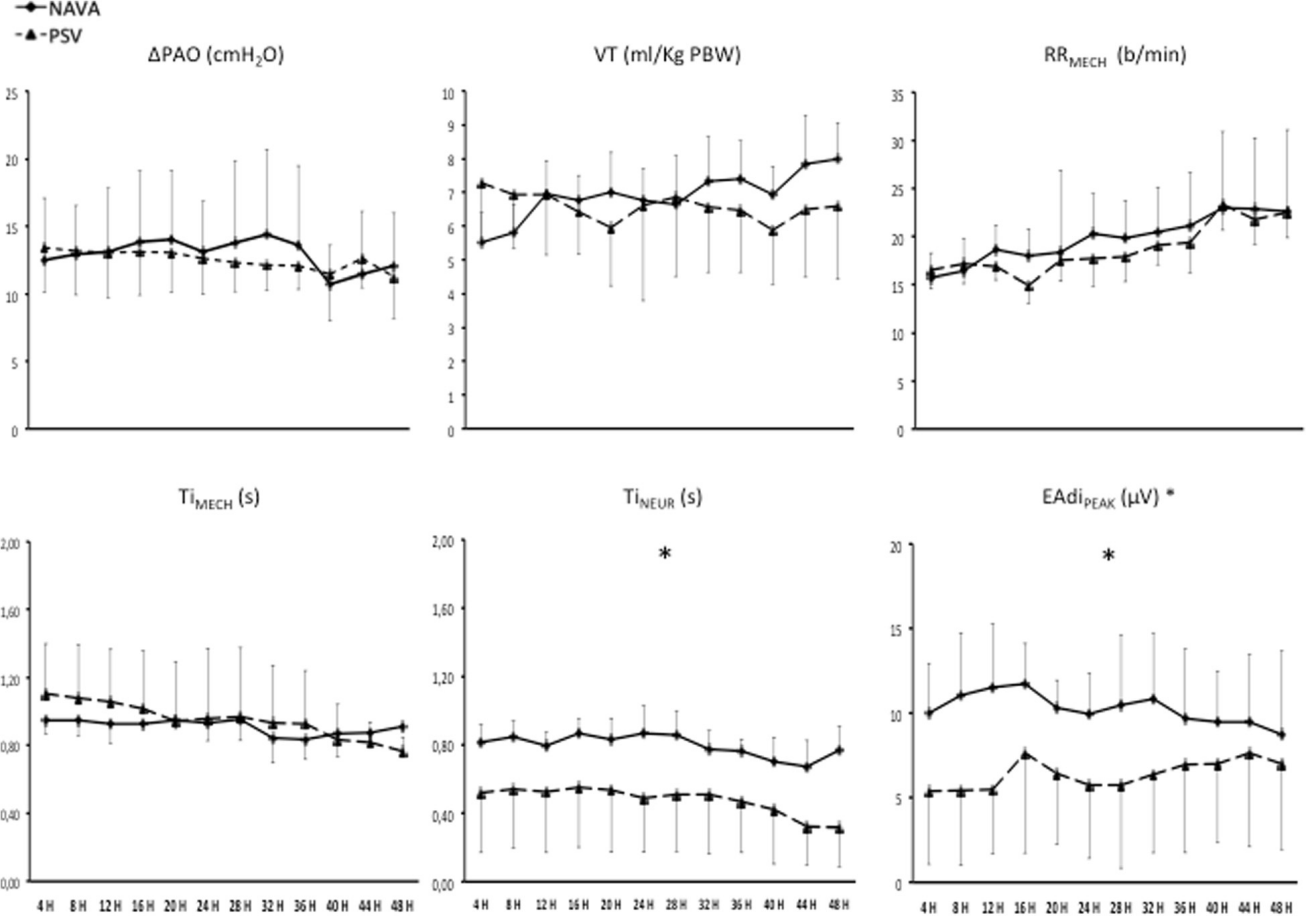
Main breathing pattern and diaphragm electrical activity (EAdi) parameters recorded each 4 hours in the two groups of patients, ventilated in neurally adjusted ventilatory assist (*NAVA*) or pressure support ventilation (*PSV*). The neural inspiratory time (*Ti*_*NEUR*_) was significantly lower in the PSV than in the NAVA group (*F* = 9.85; *p* = 0,007). The peak diaphragm electrical activity (EAdi_PEAK_) was significantly higher in the NAVA than in the PSV group (*F* = 4.83; *p* = 0,045). *Significant difference between PSV and NAVA (analysis of variance model). *ΔP*_*AO*_ assistance level (peak airway pressure – positive end-expiratory airway pressure), *VT* tidal volume, *PBW* predicted body weight, *RR*_*MECH*_ mechanical respiratory rate, *Ti*_*MECH*_ mechanical inspiratory time

Figure 3 shows the main breathing pattern and EAdi parameters recorded every 4 hours throughout the study. There was no difference between the groups for applied pressure (ΔP_AO_ = P_AO,PEAK_ – PEEP), VT and RR_MECH_. The Ti_MECH_ was similar between the two groups (average 0.91 ± 0.05 s in NAVA and 0.95 ± 0.2 s in PSV). The Ti_NEUR_ was significantly lower in the PSV than in the NAVA group (average 0.47 ± 0.30 s in PSV and 0.82 ± 0.09 s in NAVA). Accordingly, the expiratory trigger delay (Ti_MECH_ – Ti_NEUR_) was significantly higher in PSV than in NAVA (average 0.48 ± 0.05 s in PSV and 0.11 ± 0.04 s in NAVA). The EAdi_PEAK_ was significantly higher in the NAVA than in the PSV group (average 10.3 ± 2.3 μV in NAVA and 6.4 ± 4.4 μV in PSV).

Table 3 shows the total number of asynchrony events and the asynchrony index (AI) in the two groups. In the NAVA group there were significantly fewer missed efforts and prolonged cycles. The AI was significantly higher in PSV than in NAVA.

**Table 3:** Main asynchronies and asynchrony index in the two study groups

	PSV group	NAVA group	*P* value
Missed efforts (n.min^−1^)	1.48 (0.93–3)	0.54 (0.16–0.86)	0.007
Ineffective inspiratory triggering (n.min^−1^)	0.19 (0.04–0.28)	0.16 (0.03–0.51)	0.95
Double triggering (n.min^−1^)	0.08 (0.05–0.15)	0.16 (0.03–0.31)	0.56
Auto-triggering (n.min^−1^)	0.00 (0.00–0.00)	0.00 (0.00–0.00)	-
Prolonged cycles (n.min^−1^)	0.12 (0.02–0.3)	0.00 (0.00–0.001)	0.006
Short cycles (n.min^−1^)	0.03 (0.02–0.04)	0.03 (0.004–0.09)	1.00
Asynchrony index (%)	9.48 (6.38–21.73)	5.39 (3.78–8.36)	0.04

*PSV* pressure support ventilation, *NAVA* neurally adjusted ventilator assist

The EAdi_PEAK_ and Ti_NEUR_ variability (as expressed by the CV) were similar in the two groups whereas the CV of ΔPAO, VT, RR_MECH_ and Ti_MECH_ were significantly higher in NAVA than in PSV (Table 4).

**Table 4:** Coefficient of variability of the main breathing pattern parameters

	PSV group	NAVA group	*P* value
ΔP_AO_ CV (%)	0.88 (0.59–1.22)	9.32 (5.02–11.2)	0.007
VT CV (%)	7.94 (6.44–9.04)	12.83 (10.72–15.45)	0.003
RR CV (%)	10.43 (8.53–13.21)	16.31 (13.75–20.12)	0.007
Ti_MECH_ CV (%)	8.14 (7.32–8.8)	12.47 (11.45–15.45)	0.0003
Ti_NEUR_ CV (%)	11.06 (4.56–11.97)	11.05 (8.05–13.25)	0.46
EAdi_PEAK_ CV (%)	18.37 (7.87–19.56)	16.05 (15.44–20.71)	1.00

*CV* coefficient of variability, *PSV* pressure support ventilation, *NAVA* neurally adjusted ventilator assist, *ΔP*_*AO*_ assistance level (peak airway pressure – positive end-expiratory airway pressure), *VT* tidal volume, *Ti*_*MECH*_ mechanical inspiratory time, *Ti*_*NEUR*_ neural inspiratory time, *EAdi*_*PEAK*_ peak diaphragm electrical activity

**Fig. 4:**
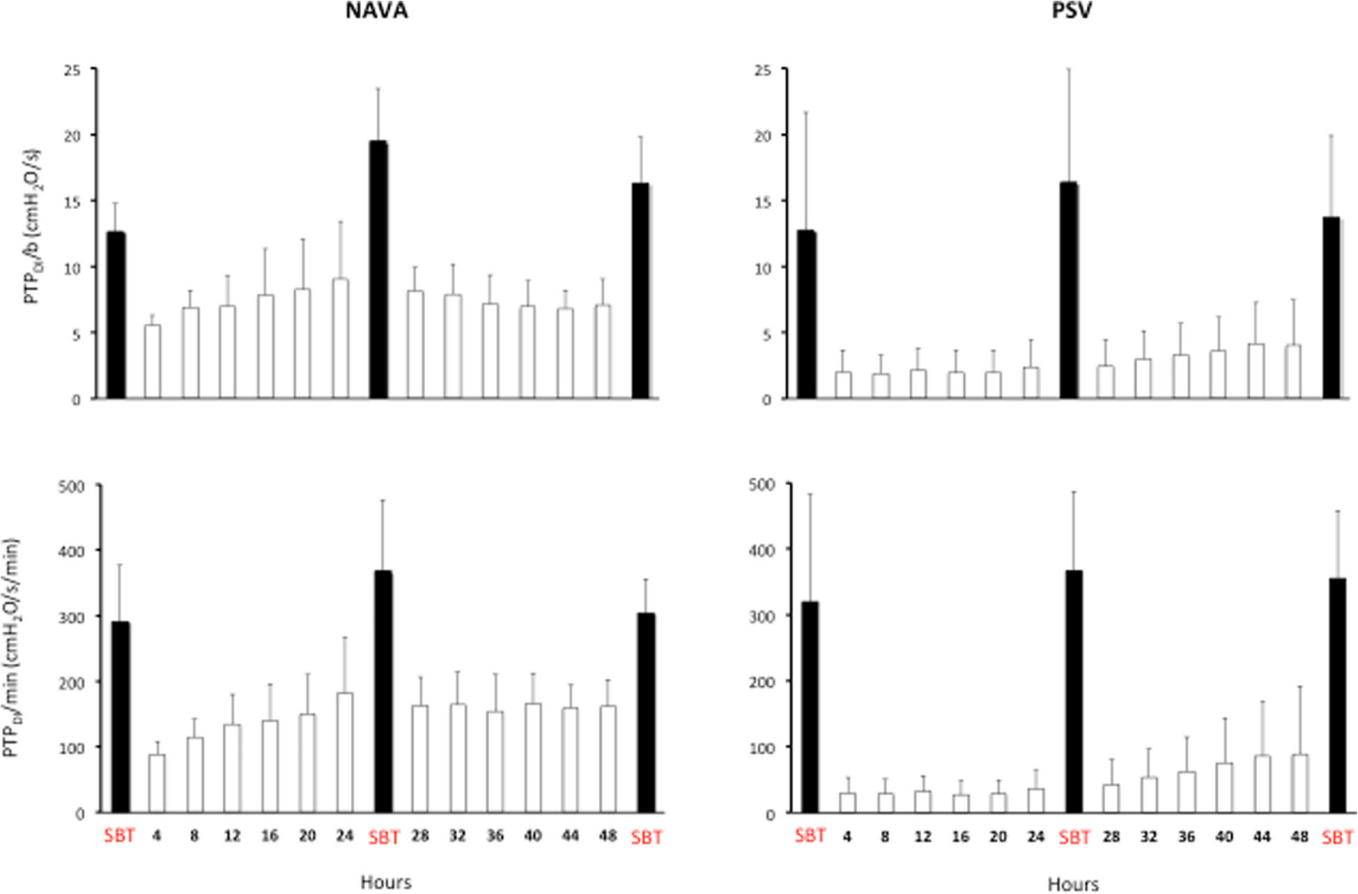
Inspiratory pressure–time product of the diaphragm (*PTP*_*DI*_) per breath (*PTP*_*DI*_ */b*) and per minute (*PTP*_*DI*_ */min*) in the two groups of patients, ventilated in neurally adjusted ventilatory assist (*NAVA*) or pressure support ventilation (*PSV*). *Black bars* denote PTP_DI_/b and PTP_DI_/minute measured during the three spontaneous breathing trials (*SBT*) (0, 24 and 48 hours). *White bars* denote PTP_DI_/b and PTP_DI_/min measured each 4 hours during assisted ventilation. During the assisted ventilation period both the P_MUSC_/b and the P_MUSC_/min were constantly higher in the NAVA than in the PSV group (analysis of variance model: for P_DI_/b, *F* = 32.64; *p* <0,001; for P_MUSC_/min, *F* = 39.15; *p* <0.001)

Figure 4 shows the PTP_DI_/b and PTP_DI_/min recorded during the three SBTs (black bars) and every 4 hours during assisted ventilation (white bars). During assisted ventilation both PTP_DI_/b and PTP_DI_/min were significantly higher in the NAVA than in the PSV group (average PTP_DI_/b 7,4 ± 1 cmH_2_O/s in NAVA and 2,7 ± 1,9 cmH_2_O/s in PSV; average PTP_DI_/min 147 ± 21 cmH_2_O/s/min in NAVA and 49 ± 39 cmH_2_O/s/min in PSV).

## Discussion

After a period of CMV potentially sufficient to decrease diaphragm efficiency [2–4, 22], we randomly assigned patients to NAVA or PSV for 48 hours. Confirming our hypothesis, in patients randomized to NAVA, two indices of diaphragmatic efficiency, NVE and NME, progressively improved, whereas they remained unchanged in patients randomized to PSV.

Electromyography (EMG) detects the translation of the neural impulse into muscle fiber action potentials. The EAdi is a processed diaphragmatic EMG recorded by an array of eight electrode pairs mounted in the wall of a nasogastric tube [15]. The temporal and spatial sum of the EMG potentials recorded by each electrode pair is converted into a single amplitude/time signal by the NAVA software (Maquet, Solna, SW) [31]. As recently reviewed by Doorduin and coworkers, the EAdi is a promising tool to monitor patient-ventilator interactions and respiratory muscle unloading during assisted ventilation [32]. In this study we used the EAdi to assess NVE and NME [19, 20, 33] and to estimate P_DI_, using a method recently validated by Bellani and coworkers [25]. Overall, our data support the idea that EAdi is a suitable tool to monitor diaphragmatic function in critically ill patients. However, we believe that further confirmatory studies are needed to standardize the EAdi measurement.

A critical issue when comparing two assisted modes is how the assistance level is set. We titrated the PSV level to obtain a VT between 5 and 8 ml/Kg PBW. This classical approach [19, 28, 34] is based on the physiological observation that excessive PSV levels, able to virtually suppress spontaneous inspiratory activity, induce a breathing pattern characterized by VTs higher than 8–10 ml/PBW and RRs lower than 15–20 breaths/minute [10, 35]. On the other hand, insufficient PSV levels are associated with low VTs (i.e., lower than 3–5 ml/Kg PBW) and high RRs (i.e., higher than 35–40 breaths/minute) [9, 10, 35]. Setting the optimal NAVA level is even more challenging [16]. Among the different approaches [28, 36, 37], we adopted the one proposed by Brander and coworkers [29, 30] that consists of a stepwise NAVA titration, while monitoring the P_AO_ and VT trend. This method is based on the evidence that, during NAVA, once the patient’s inspiratory demand is adequately supported, P_AO_ and VT fail to increase when the NAVA level is further increased [16, 38]. It may be argued that standardizing the work of breathing between NAVA and PSV would have been more appropriate for our physiological comparison [39, 40]. However, we preferred to gain physiological information on the real-life application of PSV and NAVA.

**Fig. 5:**
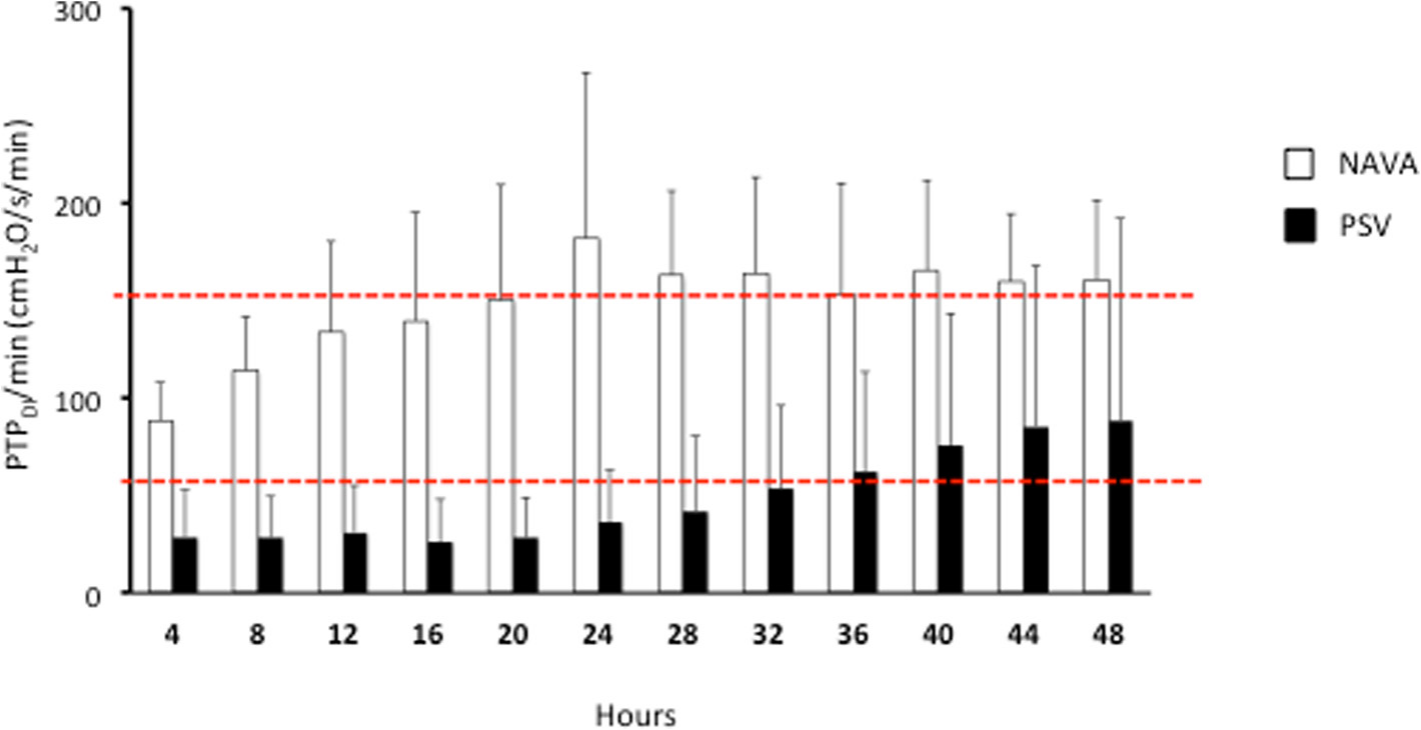
Inspiratory pressure–time product of the diaphragm per minute (*PTP*_*DI*_ */min*) in the two groups of patients, ventilated in neurally adjusted ventilatory assist (*NAVA*) or pressure support ventilation (*PSV*). White bars denote the PTP_DI_/min recorded each 4 hours during NAVA; black bars denote the PTP_DI_/min recorded each 4 hours during PSV. *Dotted red lines* denote the physiological PTP_DI_/min range according to a previous publication [42]

Respiratory muscle unloading depends on the interplay between the positive pressure applied by the ventilator, patient’s inspiratory effort and mechanical load. The inspiratory PTP_DI_ is a surrogate for work of breathing that correlates with the respiratory muscles oxygen consumption [41]. In our patients, both PTP_DI_/b and PTP_DI_/min were significantly higher in NAVA than in PSV. Figure 5 shows the PTP_DI_/min recorded in the two groups of patients during assisted ventilation, compared with the physiological PTP_DI_/min range [42]: patients in the PSV group were over-assisted for most of the study period whereas patients in the NAVA group were properly or slightly under-assisted. We speculate that over-assistance may explain why NVE and NME did not increase after 48 hours of PSV.

Our data suggest that trusting solely the breathing pattern to infer the correctness of the assistance, as commonly done in clinical practice, may be misleading. Indeed, during PSV, in spite of a frank over-assistance, our patients maintained a clinically acceptable breathing pattern for most of the study period (i.e., VT between 5 and 8 ml/Kg PBW and RR between 15 and 30 breaths/minute, Fig. 3). Consequently, our data strongly suggest that diaphragmatic activity should be continuously monitored during assisted ventilation.

In concurrence with recent reports [17–19], we found a significant higher number of missed efforts and prolonged cycles in PSV than in NAVA (Table 3). Furthermore, confirming recent data by Yonis and coworkers [18], in our patients the AI was significantly higher in PSV as compared to NAVA. These results are important in view of recent reports showing the correlation of the AI with clinically meaningful outcome parameters [13, 14]. Over-assistance and discrepancy between Ti_MECH_ and Ti_NEUR_ are major determinants of patient-ventilator asynchronies [12, 43]. As discussed above, our patients were over-assisted in PSV as compared to NAVA (Fig. 5). Furthermore, the diaphragm was passive for approximately half of the Ti_MECH_ during PSV while Ti_MECH_ and Ti_NEUR_ were remarkably similar during NAVA (Fig. 3). Even when performed by experts, the asynchrony detection based solely on the P_AO_ and Flow/time tracings (i.e., on the waveforms commonly available in the ventilator screen) is affected by a considerable inter-observer variability [44]. However, Colombo and coworkers recently showed that if also the EAdi/time tracing is available, the inter-observer variability, at least among experts in patient-ventilator interactions, becomes nil [44]. Those authors considered this method the gold standard for asynchrony detection [44]. In the present study, the visual inspection of the P_A_O, flow and EAdi waveforms was performed by three of the authors (SG, SS, CAV) with specific expertise in the field of patient-ventilator interactions. Confirming the Colombo data, their agreement in all instances was 100 %.

In our patients both EAdi and Ti_NEUR_ variability were similar during PSV and NAVA, suggesting a similar variability in the neural respiratory drive [45, 46]. However, the ability to convert neural variability into breathing pattern variability was higher in NAVA than in PSV (Table 4). Breathing pattern variability is a sign of adequate balance between respiratory muscle load and ventilatory assistance [47–49]. We speculate that this could further explain the success of NAVA in improving diaphragmatic efficiency.

As recently shown by Vaschetto and coworkers [50], during assisted ventilation, the sedation level significantly impacts on the spontaneous work of breathing, EAdi, patient-ventilator asynchronies and breathing pattern variability. However, in our study the sedation level was similar in the two groups, i.e., RASS score between 0 and –1 (Table 2). Accordingly, it is unlikely that different sedation levels could explain the differences in patient-ventilator interactions between the two groups.

To the best of our knowledge, most of the physiological studies of assisted ventilation focus on limited time periods. We, on the contrary, assessed breathing pattern, asynchronies and EAdi over a 48-hour period. As patient-ventilator interactions change over time, this is strength of our study. On the other hand, we must acknowledge the following study limitations. First, despite contribution of the diaphragm to more than 75 % of the overall work of breathing [51], NVE and NME could have been influenced by the contraction of accessory inspiratory muscles. However, we paid particular attention to exclude patients from the study who had paradoxical abdominal motion or other signs of accessory muscle contraction during the SBTs. Second, NVE is influenced both by the diaphragmatic efficiency and the mechanical load posed on the diaphragm. Thus, we cannot exclude that any modification in respiratory mechanics throughout the study could have biased the NVE trend. Third, our physiological data were obtained in a selected cohort of patients and need extra caution if extrapolated to other clinical contexts. Fourth, and most important, we found that in 7 out of 20 patients (35 %), the NAVA algorithm failed for technical reasons. Those patients were dropped from the study for loss of EAdi-pneumatic synchrony (i.e., loss of the coupling between the diaphragmatic electrical activity and ventilator pneumatic assistance) or excessively low EAdi activity. Of note, our group is experienced in the clinical application of NAVA. Thus, our findings suggest caution when applying NAVA in the clinical context.

## Conclusions

In conclusion, our data suggest that the NAVA algorithm favors diaphragm reconditioning after prolonged CMV. However, NAVA failed in 35 % of the patients for technical reasons. We found that in a prolonged observation period, patient-ventilator interactions in NAVA were superior to PSV in terms of matching between neural and mechanical inspiratory time, patient-ventilator asynchronies and breathing pattern variability. Patients were frankly over-assisted in PSV and adequately or slightly under-assisted in NAVA. Further studies are needed to assess whether the favorable physiological impact of NAVA on diaphragmatic efficiency may influence the clinical outcome.

## Key messages

- After prolonged controlled mechanical ventilation (i.e., more than 72 hours), prolonged (i.e., 48 hours) NAVA improves diaphragm efficiency whereas prolonged PSV does not
- Over a prolonged observation period, patient-ventilator interactions were significantly different between NAVA and PSV. These differences likely explain the different impact on diaphragmatic efficiency of the two techniques
- NAVA was superior to PSV in terms of matching between neural and mechanical inspiratory time, patient-ventilator asynchronies and breathing pattern variability. Patients were frankly over-assisted in PSV and adequately or slightly under-assisted in NAVA
- In the present study the EAdi signal was used to assess diaphragmatic efficiency, patient-ventilator asynchronies and patient’s inspiratory effort. Overall, our data support the idea that EAdi is a suitable tool to monitor diaphragmatic function in the critically ill patients

## Abbreviations

AI: asynchrony index
ANOVA: analysis of variance
CMV: controlled mechanical ventilation
CPAP: continuous positive airway pressure
CV: coefficient of variation
EAdi: electrical activity of the diaphragm
EAdi_PEAK_: peak electrical activity of the diaphragm
EMG: electromyography
FiO_2_: inspiratory oxygen fraction
NAVA: neurally adjusted ventilatory assist
NME: neuro-muscular efficiency
NVE: neuro-ventilatory efficiency
P_AO_: pressure airway opening
P_AO PEAK_: peak airway opening pressure
PBW: predicted body weight
P_DI_: pressure generated by the diaphragm
PEEP: positive end-expiratory pressure
P_ES_: esophageal pressure
PSV: pressure support ventilation
PTP_DI_/b: pressure–time product of the pressure generated by the diaphragm, per breath
PTP_DI_/min: pressure–time product of the pressure generated by the diaphragm, per minute
RASS: Richmond agitation sedation scale
RR_MECH_: mechanical respiratory rate
SBT: spontaneous breathing trial
SD: standard deviation
Te,_MECH_: mechanical expiratory time
Ti,_MECH_: mechanical inspiratory time
Ti,_NEUR_: neural inspiratory time
VT: tidal volume
